# Nonneural granular cell tumor treated with Mohs micrographic surgery

**DOI:** 10.1016/j.jdcr.2024.07.035

**Published:** 2024-08-28

**Authors:** Frank Z. Jing, Elliott H. Campbell, Clark C. Otley, Carilyn N. Wieland, Nahid Y. Vidal

**Affiliations:** a Department of Dermatology, Mayo Clinic, Rochester, Minnesota; b Division of Dermatologic Surgery, Department of Dermatology, Mayo Clinic, Rochester, Minnesota; c Division of Dermatopathology, Department of Dermatology, Mayo Clinic, Rochester, Minnesota

**Keywords:** benign, granular cell tumor, immunohistochemistry, malignant, Mohs micrographic surgery, nonneural granular cell tumor, tumor

## Introduction

Nonneural granular cell tumor (NNGCT) is a rare neoplasm first described in 1991 by LeBoit et al.^1^ NNGCTs differ immunohistochemically from classic granular cell tumors (GCT), lacking expression of markers of neural differentiation (eg, S-100 and SOX-10). The histopathologic characteristics suggest nonneural differentiation; however, the precise lineage of NNGCT is currently not clear. Unlike GCT which is considered benign, NNGCT typically has benign behavior but can exhibit greater nuclear atypia and mitotic activity, with potential for lymph node metastasis.^2^ Genetic analysis can demonstrate gene fusions involving the anaplastic lymphoma kinase gene.^3^ Here, we report a rare case of leukocyte common antigen-positive NNGCT successfully treated with Mohs micrographic surgery (MMS).

## Case report

**Fig 1:**
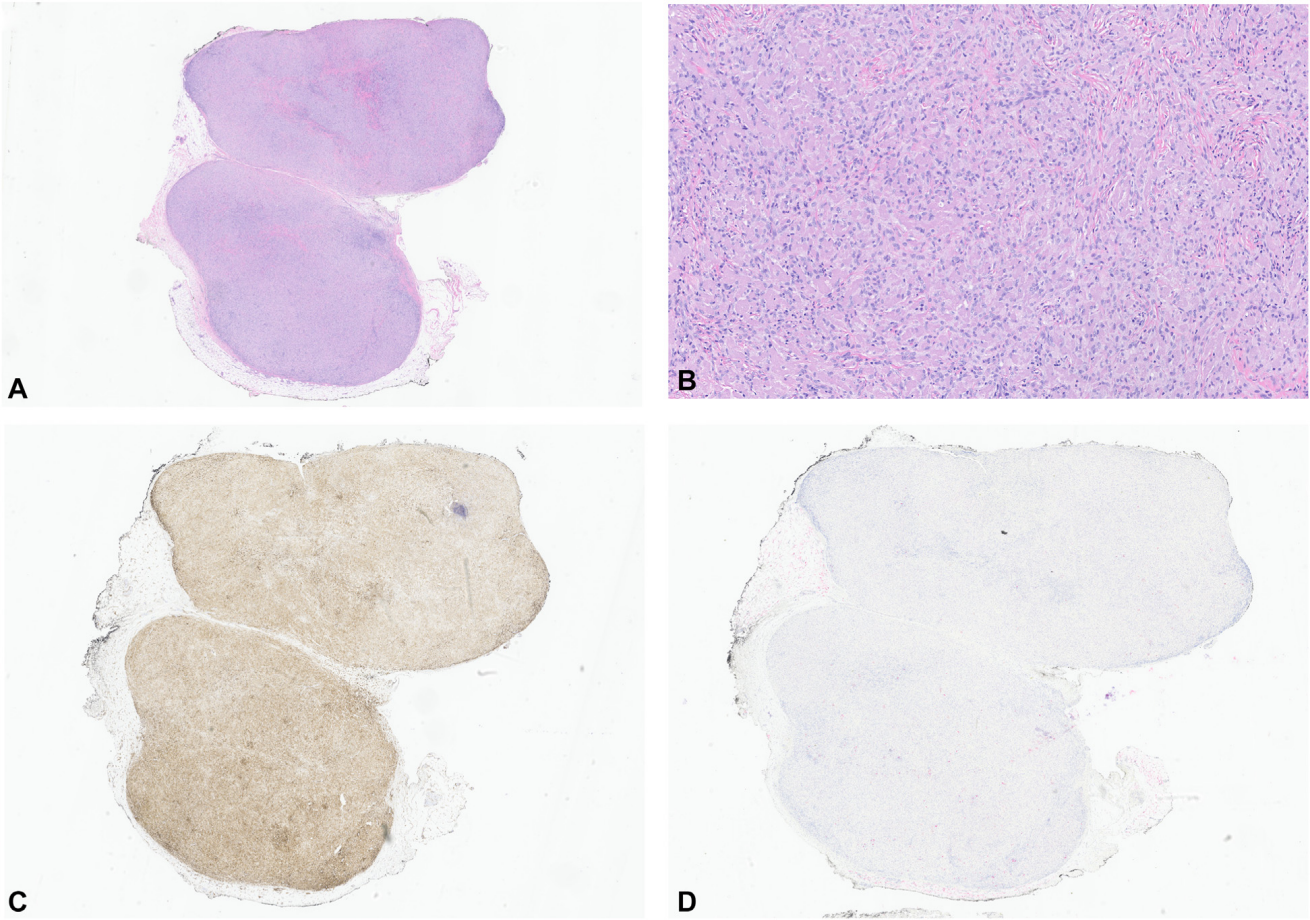
**A, B,** Hematoxylin and eosin stain of outside excision nonneural granular cell tumor specimen; **C,** diffusely positive leukocyte common antigen (CD45) staining; **D,** diffusely negative S-100 staining. (Original magnifications: **A, C,** and **D,** ×10; **B,** ×100.)

A 36-year-old woman with a history of multiple atypical nevi presented to an outside hospital for evaluation of a tender 1 cm subcutaneous nodule on the upper portion of the left arm. Three years prior, a 4 mm papule in the same location was biopsied and pathology and showed Spitz nevus with moderate to severe atypia. It was excised with pathology showing scar and no residual tumor. An excisional biopsy was performed of the new 1 cm subcutaneous nodule which revealed numerous oval and epithelioid cells with associated lymphocytes. The large epithelioid histiocytes were found to be negative for S-100, SOX-10, adipophilin, CD1a, keratin A1/A3, and CD30; and were positive for CD10, CD68, leukocyte common antigen, and factor XIIIa (Fig 1, *A-D*). Overall, the findings were consistent with cutaneous NNGCT and complete excision of the lesion was recommended.

**Fig 2:**
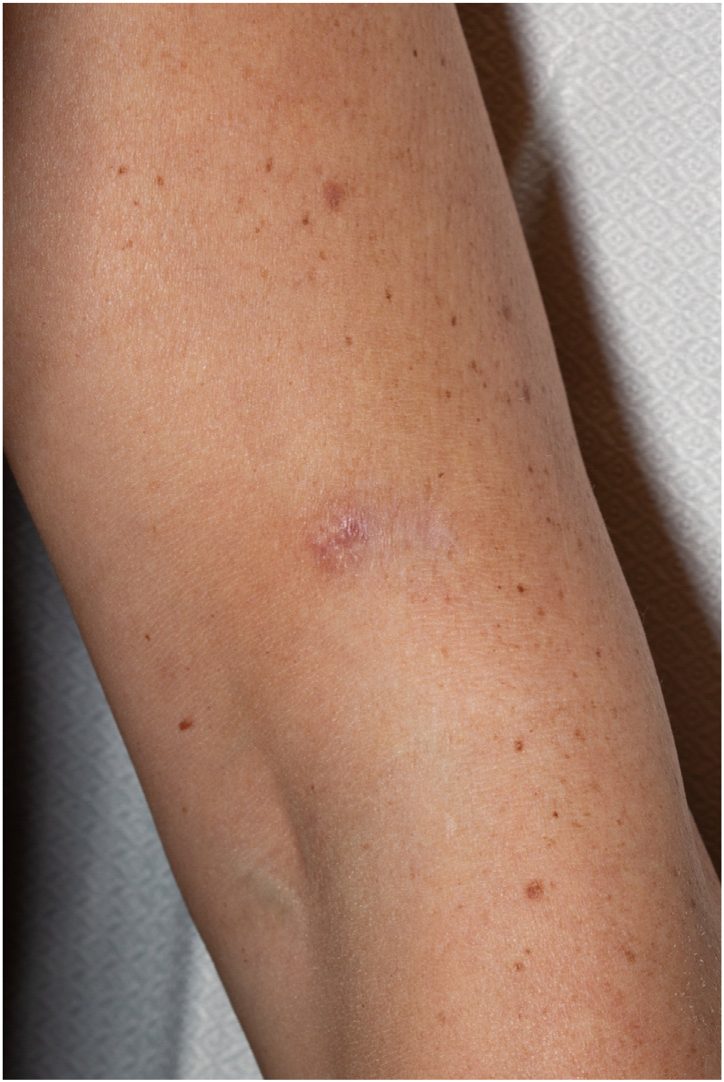
Patient presented to our institution with diagnosis of nonneural granular cell tumor and positive margins from excisional biopsy. Photograph reveals a 2 cm background hypopigmented linear scar from 3 years prior and a superimposed erythematous 1 cm scar medially from excisional biopsy.

**Fig 3:**
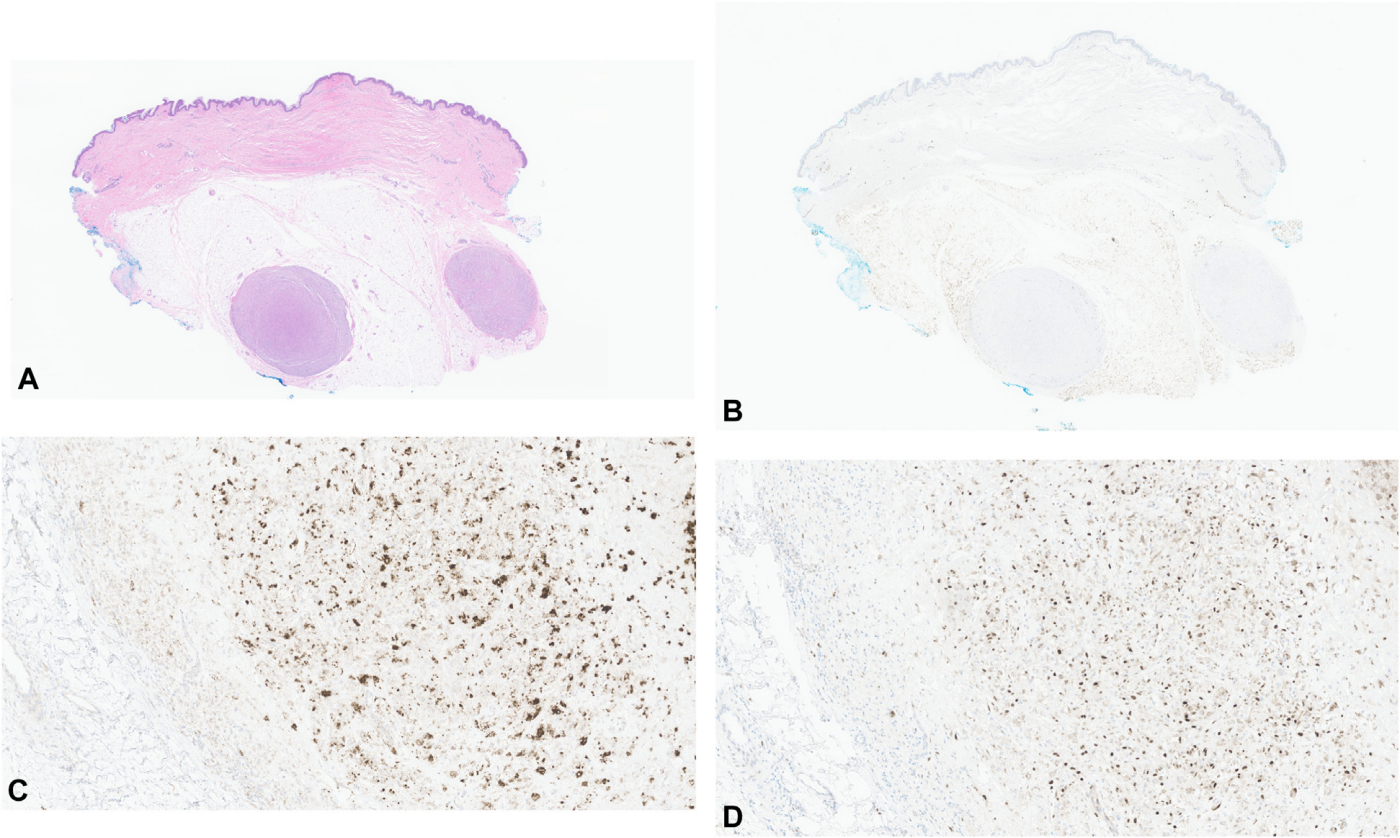
**A,** Hematoxylin and eosin stain of subcutaneous-based nonneural granular cell tumor excision specimen; **B,** diffusely negative S-100 stain; **C,** positivity for anaplastic lymphoma kinase staining; **D,** positivity for cyclin D1 staining. (Original magnifications: **A** and **B,** ×10; **C** and **D,** ×100.)

**Fig 4:**
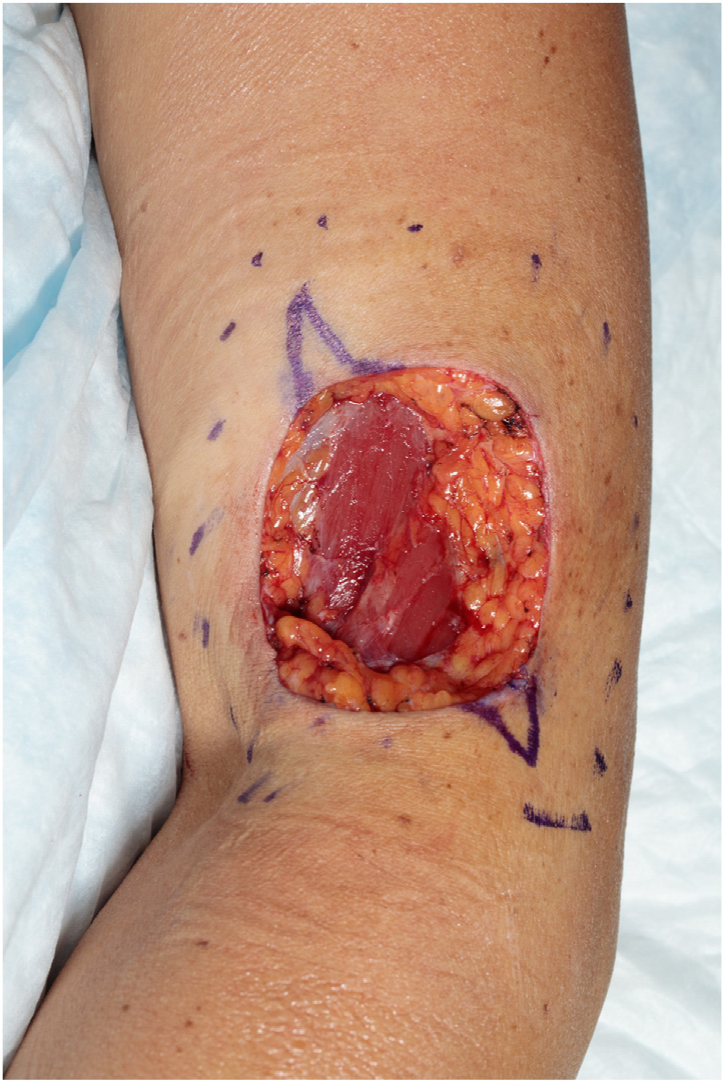
Mohs micrographic surgery final defect.

The patient presented to our institution for excision 6 months later with a well-healed 2 cm scar and more recent erythematous 1 cm scar medially, but no clinically apparent residual tumor (Fig 2). The scar was excised with 6 mm margins down to the deep subcutaneous fat with final scar length 5.6 cm. The deep and peripheral margins were positive after excision, however, with positive anaplastic lymphoma kinase and cyclin D1 immunohistochemical staining (Fig 3, *A-D*). The patient was referred for MMS to ensure complete removal. Given rarity of NNGCT, reports of malignant behavior with metastasis, and minimal available literature on staging, along with the unexpected positive margins, positron emission tomography-computed tomography was performed before MMS and negative. After the first stage of MMS, frozen tissue sections revealed questionable residual tumor admixed with inflammation; these were reviewed with a board-certified dermatopathologist who agreed. The inconclusive findings prompted a subsequent safety stage, which demonstrated unequivocally tumor-free margins, with the final defect down to muscle depth (Fig 4). The final postoperative defect size was 5.8 × 4.1 cm, which was repaired by complex layered closure with final length 10 cm. At 9-month follow-up, there were no clinical signs of tumor recurrence; an incidental computed tomography abdomen/pelvis for abdominal symptoms revealed normal findings.

## Discussion

Since the initial description of NNGCT in 1991, approximately 76 cases have been documented, with the most extensive case series to date comprising 13 patients. NNGCT manifests across all age groups but with a predilection for adolescents and young adults as the mean and median ages were 25 and 16 years, respectively.^2^ In contrast, GCT typically presents in the fourth decade of life.^3^ Cutaneous NNGCTs primarily involve the trunk and extremities, although head and neck involvement is occasionally reported.^3^^,^^4^ Clinically, NNGCTs have been described as papular, polypoid, or nodular masses and may resemble Spitz or irritated dermal nevi.

Histopathologically, NNGCTs are composed of ovoid-to-spindle cells with granular eosinophilic cytoplasm. The tumors are generally located in the dermis with well-circumscribed borders and an overlying epidermal collarette.^4^ Pustule-ovoid bodies of Milian can be identified as well. Immunohistochemical studies highlight differences between GCT and NNGCT, with the former expressing S-100 protein, NGFR-5, MBP, and/or NSE, whereas the latter is negative for S-100. In 2020, Toree-Castro et al^5^ reported 3 NNGCTs positive for cyclin D1, suggesting this marker may aid in confirming the diagnosis. Cyclin D1 positivity at present continues to be scarcely reported in literature and our case serves as an addition substantiating its usage for ambiguous cases. A rare component of this case was immunohistochemical studies returning positive for leukocyte common antigen or CD45.

NNGCTs are typically characterized by benign behavior. However, cell spindling, prominent nucleoli, high mitotic rates, and necrosis have been observed, and these features are present more frequently than in their GCT counterparts. Regional lymph node metastasis and 3 presumably malignant NNGCT cases have been reported previously.^2^^,^^6^, ^7^, ^8^ Because sentinel node biopsy is not currently standard practice, the percentage of metastatic disease in these cases is unknown and potentially underreported.

The management of GCTs, irrespective of benign or malignant nature, involves complete excision followed by close surveillance for reoccurrence.^9^ NNGCT have shown the capacity to extend beyond the dermis, infiltrating into adjacent eccrine coils, making complete clearance challenging.^10^ Although smaller and superficial lesions can typically be addressed with wide local excision, larger and deeper lesions may be more successfully treated with MMS to ensure complete removal, as in our case. Given the tumor’s proclivity for recurrence in cases with positive margins, margin control is crucial, and MMS is especially useful in functional and cosmetically sensitive areas.

## Conflicts of interest

None disclosed.
